# Integrating brain function and structure in the study of the human attentional networks: a functionnectome study

**DOI:** 10.1007/s00429-024-02824-1

**Published:** 2024-07-06

**Authors:** Mar Martín-Signes, Pedro M. Paz-Alonso, Michel Thiebaut de Schotten, Ana B. Chica

**Affiliations:** 1 Experimental Psychology Department, and Brain, Mind, and Behavior Research Centre (CIMCYC), University of Granada, Granada, 18071 Spain; 2 Groupe d’Imagerie Neurofonctionnelle, Institut des Maladies Neurodégénératives-UMR 5293, CNRS, CEA University of Bordeaux, Bordeaux, 33000 France; 3 BCBL. Basque Center on Cognition, Brain and Language, Donostia-San Sebastian, 20009 Spain; 4 Ikerbasque, Basque Foundation for Science, Bilbao, 48013 Spain; 5 Brain Connectivity and Behaviour Laboratory, Sorbonne Universities, Paris, France

**Keywords:** Attention, Phasic alerting, Spatial orienting, Executive attention, fMRI, White matter

## Abstract

Attention is a heterogeneous function theoretically divided into different systems. While functional magnetic resonance imaging (fMRI) has extensively characterized their functioning, the role of white matter in cognitive function has gained recent interest due to diffusion-weighted imaging advancements. However, most evidence relies on correlations between white matter properties and behavioral or cognitive measures. This study used a new method that combines the signal from distant voxels of fMRI images using the probability of structural connection given by high-resolution normative tractography. We analyzed three fMRI datasets with a visual perceptual task and three attentional manipulations: phasic alerting, spatial orienting, and executive attention. The phasic alerting network engaged temporal areas and their communication with frontal and parietal regions, with left hemisphere dominance. The orienting network involved bilateral fronto-parietal and midline regions communicating by association tracts and interhemispheric fibers. The executive attention network engaged a broad set of brain regions and white matter tracts connecting them, with a particular involvement of frontal areas and their connections with the rest of the brain. These results partially confirm and extend previous knowledge on the neural substrates of the attentional system, offering a more comprehensive understanding through the integration of structure and function.

**Supplementary Information:**

The online version contains supplementary material available at 10.1007/s00429-024-02824-1.

## Introduction

Attention is conceived by the majority of definitions as a mechanism for the selection of information (Posner 1994). Given that we have a limited amount of processing resources and live in a very complex world, this function is of extreme relevance. Despite being grouped under the umbrella term of attention, diverse mechanisms are employed for the selection of information. One of the most relevant models (proposed by Petersen and Posner 2012; Posner and Petersen 1990) suggested that the human attentional system can be divided into three attentional networks, namely alerting, orienting, and executive attention; with each of them representing a different set of attentional functions.

The *alerting network* (Posner and Petersen 1990; Sturm and Willmes 2001) is in charge of preparing and maintaining a state of vigilance that allows high priority signals to be processed more efficiently (faster and better). Following an external warning signal, this system can boost a transient increase in the preparation of the cognitive system (i.e., phasic alertness). Anatomically, it includes the anterior alerting system which comprises a network of midbrain and thalamic areas, as well as frontal (the anterior cingulate cortex [ACC] and the dorsolateral prefrontal cortex [dlPFC]) and inferior parietal areas (Clemens et al. 2011; Sturm et al. 1999; Sturm and Willmes 2001). In addition, tasks including warning signals activate left fronto-parietal areas (Coull et al. 2001; Fan et al. 2005; Yanaka et al. 2010) and bilateral extrastriate regions (Thiel et al., 2004).

The *orienting network* (Posner 1980; Posner and Petersen 1990), on the other hand, supports the ability to select information from a specific spatial location (spatial orienting), or from different features of objects. Spatial attention can be oriented to the location where a salient or relevant stimulus occurs, modulated by an exogenous, bottom-up, or automatic component of attention. It can also be allocated according to our plans, intentions, or task goals, modulated by an endogenous, top-down, or voluntary component of attention. Anatomically, the model of Corbetta and Shulman (2008) proposes two separate but interacting networks for spatial attention: a bilateral dorsal fronto-parietal system, including the superior parietal lobe (SPL), the intraparietal sulcus (IPS), and the frontal eye field (FEF), involved in orienting and maintaining spatial attention, related to the top-down component; and a right-lateralized ventral fronto-parietal system, including the bilateral temporoparietal junction and the right middle and inferior frontal gyri (MFG/IFG), involved in attentional re-orienting to unexpected or salient events, related to the bottom-up component (Corbetta et al., 2008; see also Thiel et al., 2004, Alves et al. 2022). Recent work has extended this cortical model of orienting of attention by highlighting the important role of subcortical structures, including the pulvinar, the superior colliculi, and the caudate nucleus (Alves et al. 2022).

The *executive attention network* is required in situations for which we do not have a learned schema of action, or our schemas are not appropriate (Petersen and Posner 2012). These are typically novel, difficult, dangerous, or changing situations, involving planning, decision-making, or the need to detect and solve conflicts or errors (Norman and Shallice 1986). A broad network of regions has been related with the executive attention network, including the ACC, the dlPFC, the supplementary motor area (SMA), the anterior insula, the premotor cortex, the posterior parietal cortex, and the inferior frontal junction (Braver 2012; Cole and Schneider 2007; Macdonald et al. 2000; Miller and Cohen 2001; Posner and Petersen 1990).

In addition to studies focused on identifying brain regions where activity is modulated by specific attentional demands, work on whole-brain functional connectivity has confirmed the idea that attention emerges from the dynamic interaction of many distinct brain regions (Rosenberg et al. 2017). These studies suggest that there could be a general “attention factor”, reflected in the brain’s intrinsic functional organization, that recruits, among others, the salience, subcortical, cerebellar, and frontoparietal networks. Such whole-brain connectome models can help to predict specific attention components in task-performance, and capture inter-individual and intra-individual variability (Kardan et al. 2022; Yoo et al. 2022).

As mentioned above, our current knowledge about the contribution of grey matter brain areas to healthy attentional functions is quite extensive, thanks to the use of functional magnetic resonance imaging (fMRI), among other neuroimaging techniques. However, evidence concerning the anatomical pathways connecting and functionally linking the attentional brain networks is much more recent and scarce. Over the last years, the development of methods such as diffusion-weighted imaging (DWI) has allowed us to investigate in vivo the structural brain connectivity and the micro- and macrostructural properties of white matter tissue. Nevertheless, to the best of our knowledge, only a few studies have examined the brain connectivity underlying the healthy human attentional networks. Moreover, the evidence from these studies has not always been consistent. The alerting network has been linked to the left posterior limb of the internal capsule (Niogi et al. 2010), the right dlPFC-caudate tract, the splenium of the corpus callosum (Luna et al. 2021), the cerebellothalamic tract (Ge et al. 2013) and the superior longitudinal fasciculus (SLF, Chica et al. 2018). Some studies have associated the orienting network with the splenium of the corpus callosum (Niogi et al. 2010) and the SLF (Carretié et al. 2012; Chica et al. 2018; Ge et al. 2013; Thiebaut de Schotten et al. 2011). Finally, the executive attention network has been related with the anterior corona radiata (Ge et al. 2013; Niogi et al. 2010) and the SLF (Crespi et al. 2018; Sasson et al. 2012, 2013; Smolker et al. 2018).

Most of the results of these DWI studies are based on correlations between different measures of white matter properties (e.g., fractional anisotropy, Le Bihan et al. 2001; or hindrance modulated orientational anisotropy, Dell’Acqua et al., 2013) and behavioral indices of attentional functions. Therefore, these investigations are of relevance to understand how relatively stable characteristics such as the microstructural properties of the relevant anatomical connections correlate with cognitive functions (tract-function correlations). However, they do not reflect the functional involvement of white matter during attentional tasks, as the existing neuroimaging methods did not allow such relationships to be established. In this vein, some recent developments have allowed the detection of task-related and resting-state white matter fMRI signals (Gawryluk et al. 2014; Gore et al. 2019). However, while very promising, this approach needs more time and practice to be fully understood and generalized. Recently, new proposals have also tried to integrate functional and structural data. The *functionnectome* approach (Nozais et al. 2021) projects the fMRI signal from grey matter voxels to the white matter, and weights the signal by the probability of structural connections, which are derived from 100 high-resolution DWI datasets. These new methods hold much promise to integrate function and structure to further understand core questions in the field of cognitive neuroscience beyond localization of function, such as how do function and structure interact? Or how does structure support cognitive function? Indeed, since the very first post-mortem anatomical dissections, the study of white matter has allowed the scientific community to move from a more localizationist approach to a more associationist approach. The development of DWI enriched the comprehension of brain lesions and neurological syndromes, allowing a better understanding of the functional models of the brain and enabling the discovery of individual variability and its relationship with healthy cognition, recovery after brain lesions, and response to neuromodulation (Assaf et al. 2019; Forkel et al. 2022). We consider the integration of function and structure to be vital to inform current models of human cognition and, in particular, to shed further light on theoretical accounts of human attentional networks.

Thus, the main aims of the present work are to examine the involvement of anatomical circuits in the functional correlates associated with the alerting, orienting, and executive attentional networks, as well as to contribute to the scarce, and sometimes inconsistent, knowledge of white matter contributions to healthy attentional functions. To this end, we have utilized a new method with high sensitivity, specificity, and reproducibility that allows the exploration of the involvement of anatomical circuits in specific cognitive functions (the *functionnectome*, Nozais et al. 2021). Based on previous studies, we hypothesized the main fronto-parietal association tract, the SLF, would be involved in all three attentional networks (Carretié et al. 2012; Chica et al. 2018; Crespi et al. 2018; Ge et al. 2013; Sasson et al. 2012, 2013; Smolker et al. 2018; Thiebaut de Schotten et al. 2011). We also expected a left lateralization of the alerting network (Fan et al. 2005; Yanaka et al. 2010) and a right lateralization of the orienting network (Thiebaut de Schotten et al. 2011). Additionally, we expected that the alerting network would recruit projection tracts, given that the anterior alerting system depends on cortico-subcortical interactions (Clemens et al. 2011; Sturm et al. 1999; Sturm and Willmes 2001). Finally, we predicted that the executive attention network would chiefly rely on white matter tracts connecting the frontal lobe with the rest of the brain.

## Methods

### Datasets

**Fig. 1:**
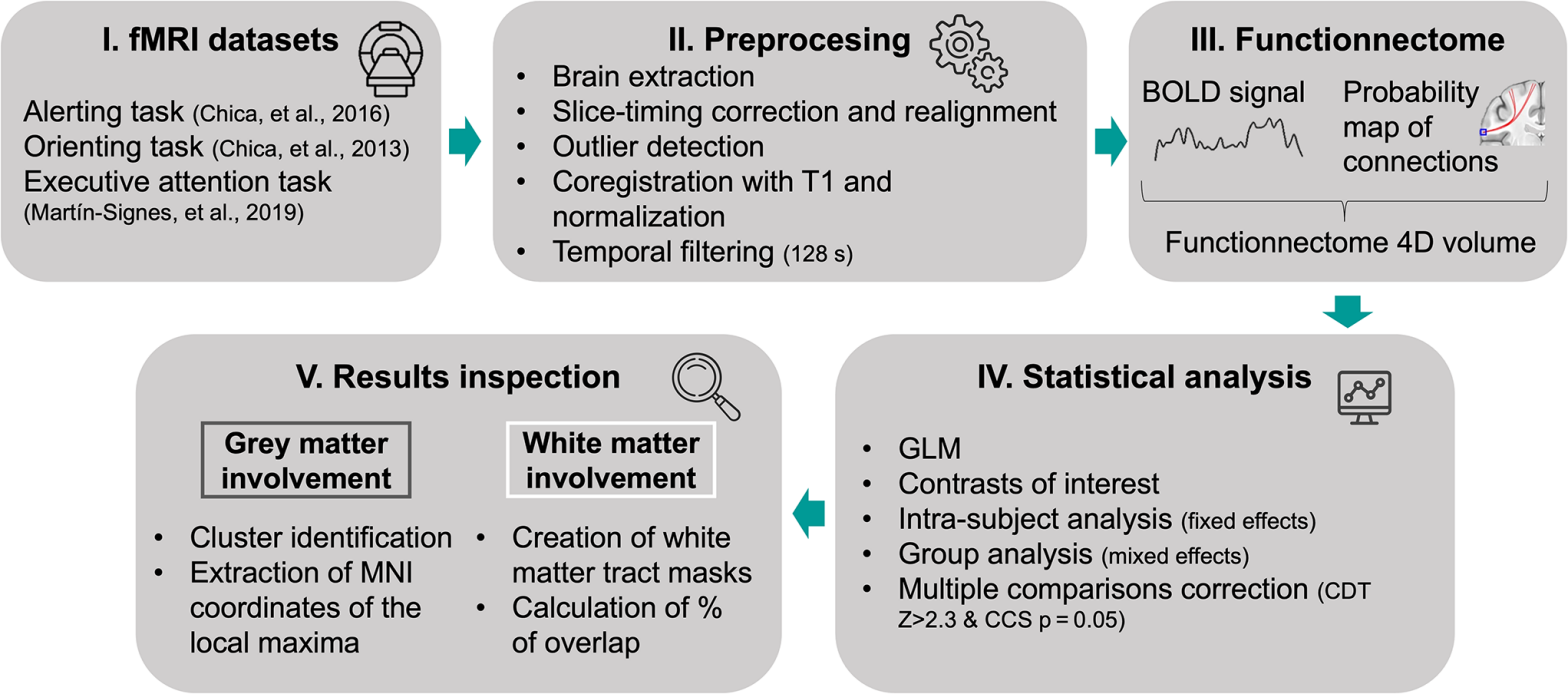
Pipeline of the fMRI procedure including key steps. CCS, cluster detection threshold; CDT, cluster-defining threshold; GLM, general linear model; MNI, Montreal Neurological Institute

Three previously collected and published datasets were employed in this study for the alerting task (18 participants, Chica et al. 2016), the orienting task (18 participants, Chica et al. 2013) and the executive attention task (20 participants, Martín-Signes et al. 2019) (Fig. 1.I). Data was originally collected with the informed consent of the participants, following the principles of the Declaration of Helsinki, and with the approval of the Ethics Committee of the INSERM (France) and the University of Granada (Spain). Datasets were provided by the authors of the original studies and analyzed with their consent.

### Experimental tasks

**Fig. 2:**
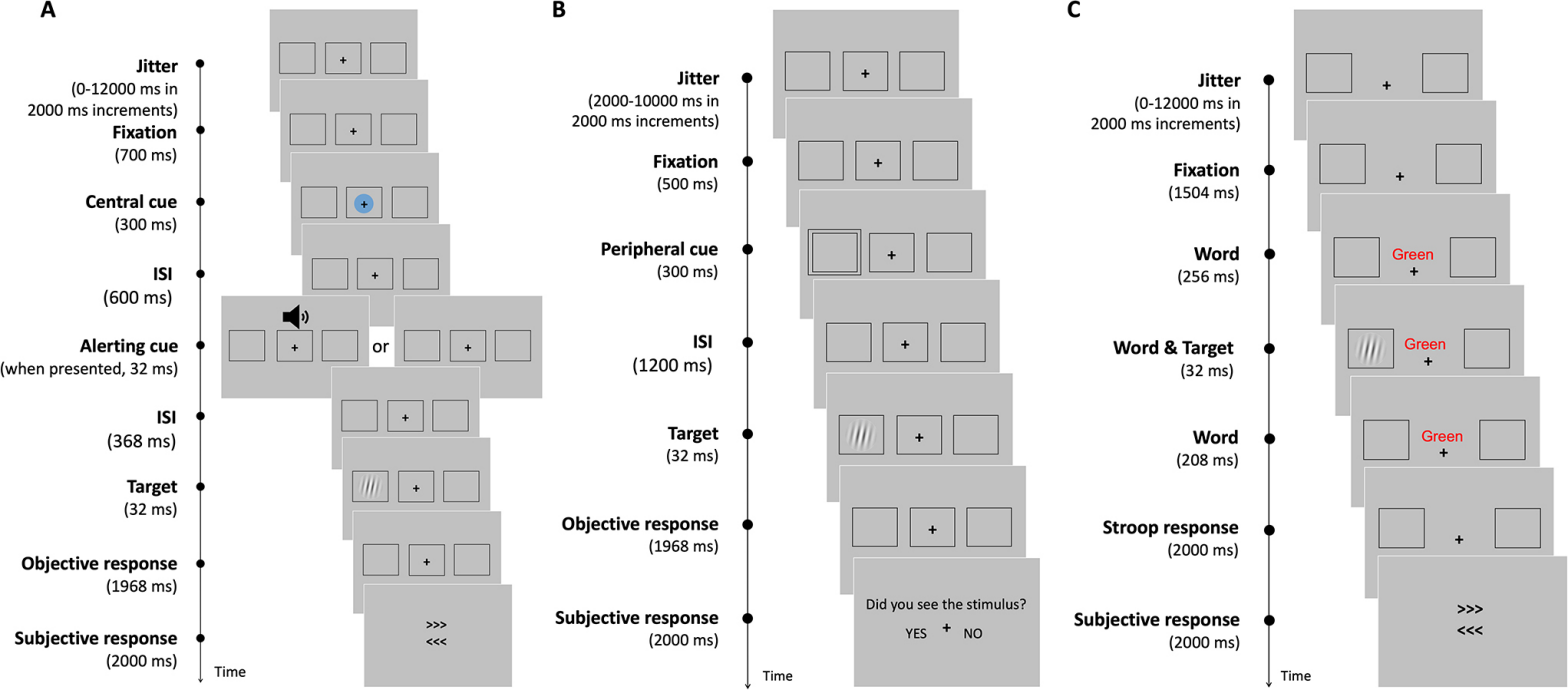
Sequence and timing of events in a given trial of each of the three attentional tasks: (**A**) alerting task, (**B**) orienting task, and (**C**) executive attention task. Adapted with permission from Chica et al. 2013, 2016; Martín-Signes et al. 2019

Full description of the stimuli and procedure can be found in the original publications (Chica et al. 2013, 2016; Martín-Signes et al. 2019). All three studies employed a visual perceptual task with three complementary attentional manipulations (i.e., phasic alerting, spatial orienting, and executive attention; see Fig. 2). The target was a near-threshold stimulus that could appear inside one of two lateral boxes (located on the right and left visual field). In some trials (catch trials) the target was not presented (these trials ranged between 13 and 25% depending on the experiment). Target contrast was manipulated for each participant before the experimental task in order to adjust the percentage of consciously perceived targets to ≈ 50%. Participants’ responses were given manually by pressing buttons on an MRI compatible fiber optic box.

For the *alerting task*, the alerting cue (white noise) was presented on 50% of the trials. The task also included a central endogenous cue but this manipulation is not analyzed in the present study. Participants were required to give two consecutive responses: first, they had to discriminate the orientation of the lines composing the target (objective response); and second, they had to report if they consciously detected its appearance (subjective response) by indicating its location (right or left box) or indicating that the target was not seen (see Fig. 2A). The experiment consisted of two sessions with 5 functional scans of 12 min duration each (920 trials in total).

For the *orienting task*, a peripheral cue was presented for 300 ms and consisted of a square surrounding one of the lateral boxes. The cue was predictive about the spatial location of the target on 67% of the target-present trials (valid trials), while the remaining 33% of the target-present trials were invalid (where the target was presented at the opposite location to the cue). Similar to the alerting task, participants were required to give an objective and a subjective response. The latter was made by responding to the question ‘‘Did you see the stimulus?’’ with a “yes” or “no” answer (see Fig. 2B). The experiment consisted of one session with 5 functional scans of 7 min duration each (280 trials in total).

For the *executive attention task*, a Stroop task was centrally presented, concurrently to the appearance of the target. Spanish words for blue, green, and yellow colors were displayed either in blue, green, or yellow color. On congruent trials the word meaning and the color matched, and on incongruent trials the word meaning and the color were different (20% of trials). Participants performed two consecutive tasks: first, they had to discriminate the word’s color, and second, they had to report if they consciously detected the appearance of the target (right or left box, or unseen target) (see Fig. 2C). The experiment consisted of 2 sessions with 5 functional scans of 8 min duration each (600 trials in total).

Images of the three attentional tasks were presented on a screen located at the back of the scanner and viewed with a mirror mounted on the head coil. The jitter fixation and the order of trial types within each scan in all three tasks were determined with an optimal sequencing program (i.e., Optseq2), designed to maximize the efficiency of recovery of the blood-oxygen-level dependent (BOLD) response ((Dale 1999); http://surfer.nmr.mgh.harvard.edu/optseq/*).*

### Acquisition parameters

Full descriptions of the acquisition parameters can be found in the original publications (Chica et al. 2013, 2016; Martín-Signes et al. 2019). Whole-brain fMRI was conducted on two different Tesla Siemens TRIO MRI scanners using a whole-head coil. Functional images were acquired using a gradient-echo echo-planar pulse sequence with the following parameters for the *alerting task* (time-to-repetition [TR] = 2000 ms, time-to-echo [TE] = 25 ms, 39 axial 3-mm cubic slices, no inter-slice gap, flip angle = 75°, field of view [FoV] = 220 mm, 372 volumes acquired per run), the *orienting task* (TR = 2000 ms, TE = 25 ms, 34 axial 2.5 × 2.5 × 3-mm slices, no inter-slice gap, flip angle = 75°, FoV = 220 mm, 220 volumes acquired per run), and the *executive attention task* (TR = 2000 ms, TE = 25 ms, 35 axial 3.4-mm cubic slices, no inter-slice gap, flip angle = 75°, FOV = 220 mm, 245 volumes per run). High-resolution T1-weighted anatomical images were also collected (TR = 2300 ms, TE = 4.2 ms, flip angle = 9º, FoV = 256 mm, voxel size = 1 × 1 × 1 mm, 176 slices, for the alerting and orienting tasks; TR = 2530 ms, TE = 3.5 ms, flip angle = 7º, FoV = 256 mm, voxel size = 1 × 1 × 1 mm, 176 slices, for the executive attention task). DWI data was only collected for one of the datasets (Martín-Signes et al. 2019). Note that DWI data from the participants’ sample was not used in the present study since the *functionnectome* employs a normative high-resolution tractography (Nozais et al. 2021). The full fMRI pipeline is illustrated in Fig. 1.

### fMRI preprocessing

Preprocessing routines and analyses were performed using FEAT (FSL, FMRIB’s Software Library, Woolrich et al. 2001) (Fig. 1.II). Brain extraction was performed using BET (Smith 2002). Images were corrected for differences in timing of slice acquisition and were realigned to the middle volume by means of rigid-body transformation for motion correction using MCFLIRT (Jenkinson et al. 2002). Motion plots were visually inspected to discard those runs with excessive motion (i.e., relative motion > than half of voxel size, or absolute motion > than voxel size). Only in the alerting experiment some runs were eliminated for exceeding these parameters. Concretely, for three participants we excluded 1 to 3 runs. Structural and functional volumes of each participant were coregistered using the Boundary-Based Registration function. Next, the structural volume was registered to a standard image and a similar transformation was applied to the functional volume using a non-linear registration with 12 degrees of freedom. During normalization, volumes were sampled to 2 mm isotropic voxels and standard images were based on the MNI152 stereotaxic space. A 128s high-pass filter was used to eliminate contamination from slow drift of signals. Outlier scans corrupted by large motion were detected using the tool *fsl_motion_outliers.* This tool detects outliers if the root-mean-squared (RMS) intensity difference to reference volume exceeds the 75th percentile + 1.5 times the InterQuartile Range. The identified outliers were regressed out from the data with a GLM where each outlier was entered as a nuisance regressor. The mean percentage of detected outlier volumes per run was 5.5% (SD = 3.12%) for the whole dataset (alerting task, M = 6.6%, SD = 3.3%; orienting task, M = 4.3%, SD = 2.8%; executive attention task, M = 5.3%, SD = 2.8%). For each task, and even for the participant with the highest proportion of data removed (which was always lower than 20%), the BOLD signal of the minimum condition of interest was 11.5, 7.1, and 6.6 min for the alerting, orienting, and executive attention tasks, respectively. No spatial smoothing was applied as the *functionnectome* method has an analogous effect of improving the signal-to-noise ratio by combining the signal from distant yet structurally linked voxels (Nozais et al. 2021).

### Functionnectome

We applied the *functionnectome* over the preprocessed data (Fig. 1.III; for a detailed description of the method see Nozais et al. 2021). This method projects the signal from each voxel of the fMRI 4D volume to the white matter according to their structural relationships. These structural relationships were based on a probability map that is composed by the structural connectivity between a given voxel and the rest of the brain. This map is derived from a normative high-resolution tractography acquired at 7T in 100 subjects from the preprocessed version of the human connectome project (raw data openly available at www.humanconnectome.org and preprocessed tractographies at https://osf.io/5zqwg/). This process generates a new 4D volume projecting the fMRI signal from grey matter voxels to the white matter, weighted by the probability of connection. This new 4D volume can be statistically analyzed similarly to a classical fMRI volume. The *functionnectome* is open-source software available at http://www.bcblab.com.

### fMRI analysis

fMRI analysis and results inspection steps are briefly illustrated in Fig. 1.IV and V. Statistical analyses were performed for each individual run using the general linear model (GLM). Task regressors were convolved with the FSL double-gamma function. For the three attentional tasks, fMRI trials were sorted as “seen” or “unseen” according to participants’ subjective responses. For the *alerting task*, we used a model including the three phases of the fMRI trial: cue presentation, target presentation (including the objective response), and subjective response. The cue presentation period was modeled as 6 regressors of interest including tone seen, tone unseen, no tone seen, no tone unseen, target-absent tone, and target-absent no tone trials. Missed responses and errors were modeled but not included in the analysis^1^. The contrast of interest for this task was defined as Tone > No tone trials.

For the *orienting task*, we used a GLM including the three phases of the fMRI trial: cue presentation, target presentation (including the objective response), and subjective response. The cue presentation period was modeled as 4 regressors of interest including valid seen, valid unseen, invalid seen, and invalid unseen trials. Missed responses, errors, and target absent trials were modeled but not included in the analysis. The contrast of interest for this task was defined as Cue trials > Null. Null consisted of the unmodeled periods (i.e., Jitter fixation). Cue trials included all cue periods (independently of their validity in relation to target location). Note that during the cue period the target had not yet appeared and therefore it was not possible to distinguish between valid and invalid cues.

For the *executive attention task*, we used a GLM including the three phases of the fMRI trial: stimuli presentation (color word and target), Stroop response, and subjective response. The stimuli presentation period was modeled as 4 variables of interest including congruent seen, congruent unseen, incongruent seen, and incongruent unseen trials. Missed responses, errors, and target absent trials were modeled but not included in the analysis. The contrast of interest for this task was defined as Incongruent > Congruent trials.

For the three tasks, to prevent motion artifacts, six head motion parameters and outlier scans were entered as regressors of no interest in all first-level analyses. Intra-subject brain activations of the contrast of interest were calculated using fixed effects. Higher-level mixed-effects were carried out using FLAME 1 (Woolrich et al. 2004), and Z-statistic BOLD images were rendered using a cluster-defining Z-threshold of > 2.3 and a corrected cluster significance threshold of *p* = 0.05.

For all contrasts of interest, we reported the Montreal Neurological Institute (MNI) coordinates of the local maxima of the grey matter regions showing significant activations inside a larger cluster. To identify and report white matter tracts involved in each attentional task, we created a mask per each of the 42 tracts included in the XTRACT probabilistic tract atlas (Warrington et al. 2020), plus the corpus callosum (genu, body, and splenium) from the JHU ICBM-DTI-81 atlas (Mori et al. 2005). These masks were applied to the contrast images to calculate the number and percentage (total number of significant voxels divided by the number of voxels of the tract mask) of voxels and the local maxima of the brain activations overlaid by the tract mask. We considered those tracts with a total number of voxels of overlap greater than the 1% of the total number of voxels of the contrast images of each experiment (i.e., alerting: 195 voxels; orienting: 57 voxels; executive attention: 645 voxels). In addition, we considered tracts where a tract mask overlapped less than 1% of the total significant voxels of the contrast but the percentage of overlap with the tract mask was greater than 25% of the tract.

In order to examine the association between brain activations and the use of attentional signals, we correlated the whole-brain contrast of interest for each task with a relevant behavioral index using a voxel-wise approach. Results were obtained using FLAME 1 and a Z-threshold of > 2.3 with a corrected cluster significance threshold of *p* = 0.05. For the alerting task, we calculated this index by subtracting the reaction time (RT) of the no tone minus the tone condition; for the orienting task, we subtracted the RT of the invalid minus the valid condition; for the executive attention task, we subtracted the RT of the incongruent minus the congruent condition. Note that the RT for the alerting and orienting tasks was calculated by the objective response to the target, while for the executive attention task the RT was calculated by the response to the Stroop word. These behavioral indices were expected to be positive on average (as slower RTs are expected in the no tone, invalid, and incongruent conditions) and higher values would indicate better use of the attentional signals (for alerting and orienting tasks) or higher impairment due to overload of the executive attention system (for the executive attention task). To calculate the effect size of the correlations between the brain signal and each behavioral index, we extracted the mean value from each significant cluster and performed a Pearson correlation analysis.

## Results

### Alerting task

**Fig. 3:**
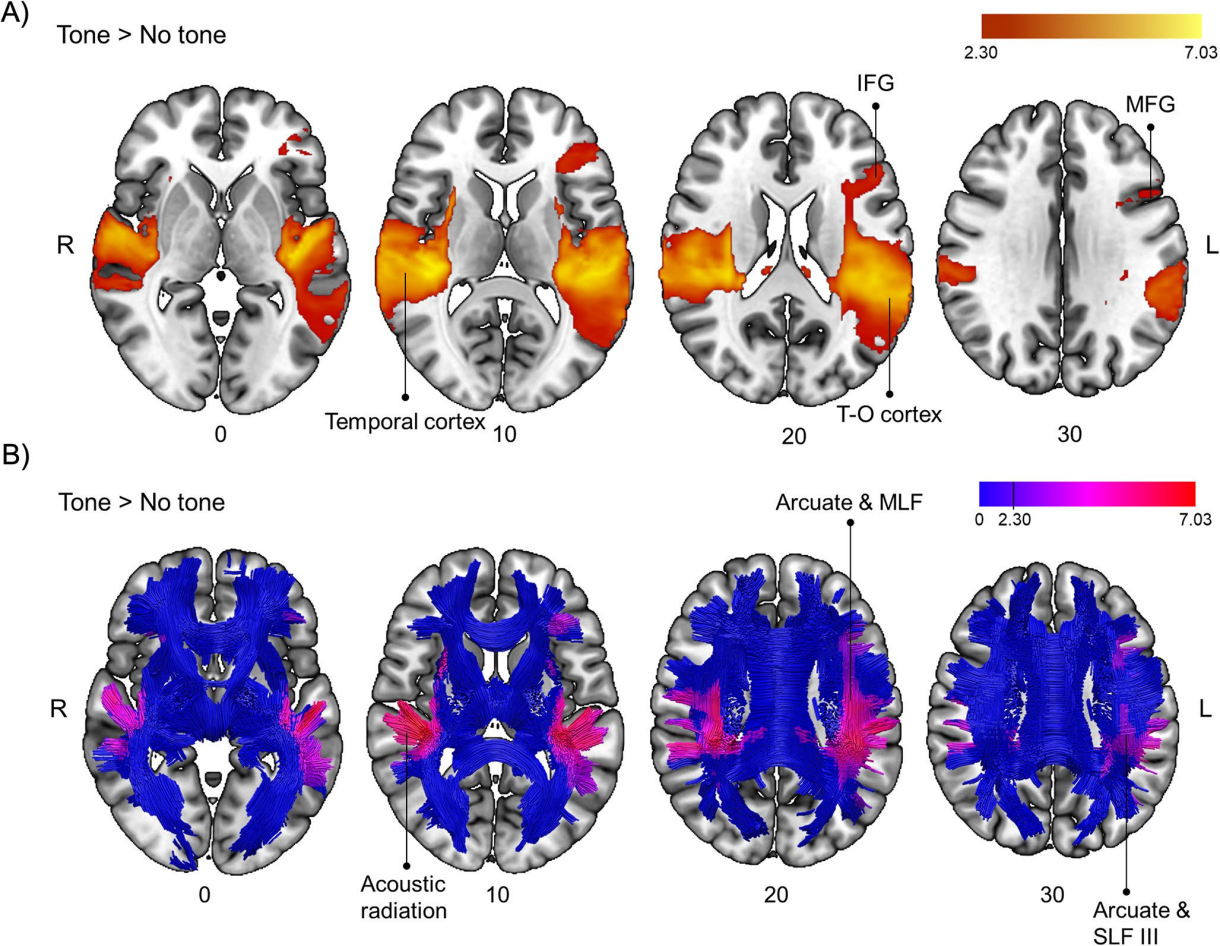
(**A**) Brain sections showing activations obtained in the contrast Tone > No tone for the alerting task at a cluster-defining threshold of Z > 2.3 and a corrected cluster significance threshold of *p* = 0.05. The color bar denotes Z-values. (**B**) Representation of activations depicted in panel A over a normalized template of tractography. Tractography slices are 10 mm thick to create a 3D effect. Blue color represents the skeleton of fibers and colors from purple to red denote Z-values. IFG, inferior frontal gyrus; MLF, middle longitudinal fasciculus; SLF III; superior longitudinal fasciculus, ventral branch; T-O cortex, temporo-occipital cortex

For the contrast Tone > No tone (see Table 1; Fig. 3), we found functional activations involving white matter association tracts connecting temporal regions with frontal (i.e., the bilateral arcuate), occipital (i.e., the left inferior longitudinal fasciculus, ILF), and occipital-parietal regions (i.e., the bilateral middle longitudinal fasciculus, MLF). In addition, we observed activations that involved white matter tracts running between frontal and occipital regions (i.e., the left inferior fronto-occipital fasciculus, IFOF) and between frontal and parietal regions (i.e., the left SLF III), and between regions within the frontal lobe (i.e., the left frontal aslant tract, FAT). Regarding white matter projection tracts, we observed significant functional activations involving the bilateral acoustic radiation and the left optic radiation. Grey matter regions involved by these connections are shown in Table 1. Additional sagittal and coronal views of the results can be found in Supplementary Fig. 1.

**Table 1:** Brain activations and connections involved in the contrast Tone > No tone for the alerting task

Functional involvement	Side	X	Y	Z	z-value	
*White matter pathways*						*Voxels of overlap*
Acoustic radiation	L	−32	−28	8	6.31	887 (61%)
	R	50	−16	6	6.80	816 (60%)
Arcuate fasciculus	L	−50	−30	10	6.68	2159 (80%)
	R	42	−30	12	7.00	1059 (33%)
Frontal aslant tract	L	−34	4	14	3.77	371 (15%)
Inferior fronto-occipital fasciculus	L	−32	−28	6	5.92	352 (10%)
Inferior longitudinal fasciculus	L	−66	−20	−18	4.13	1421 (42%)
Middle longitudinal fasciculus	L	−44	−16	−2	6.82	1513 (50%)
	R	34	−30	16	6.62	1218 (42%)
Optic radiation	L	−30	−34	10	5.27	221 (10%)
Superior longitudinal fasciculus III	L	−52	−44	20	5.72	916 (24%)
*Connections to*						*Cluster size*
Temporal lobe	L	−40	−30	14	7.03	19,486 (*)
	R	42	−30	12	7.00	19,486 (*)
Inferior frontal gyrus	L	−50	38	14	3.62	19,486 (*)
Middle frontal gyrus	L	−38	20	26	3.30	19,486 (*)
Temporo-occipital cortex	L	−56	−72	16	3.17	19,486 (*)
Fusiform gyrus	L	−42	−36	−22	3.16	19,486 (*)
Orbitofrontal cortex	R	44	26	−12	3.11	19,486 (*)

(*) These clusters belong to a larger cluster

**Fig. 4:**
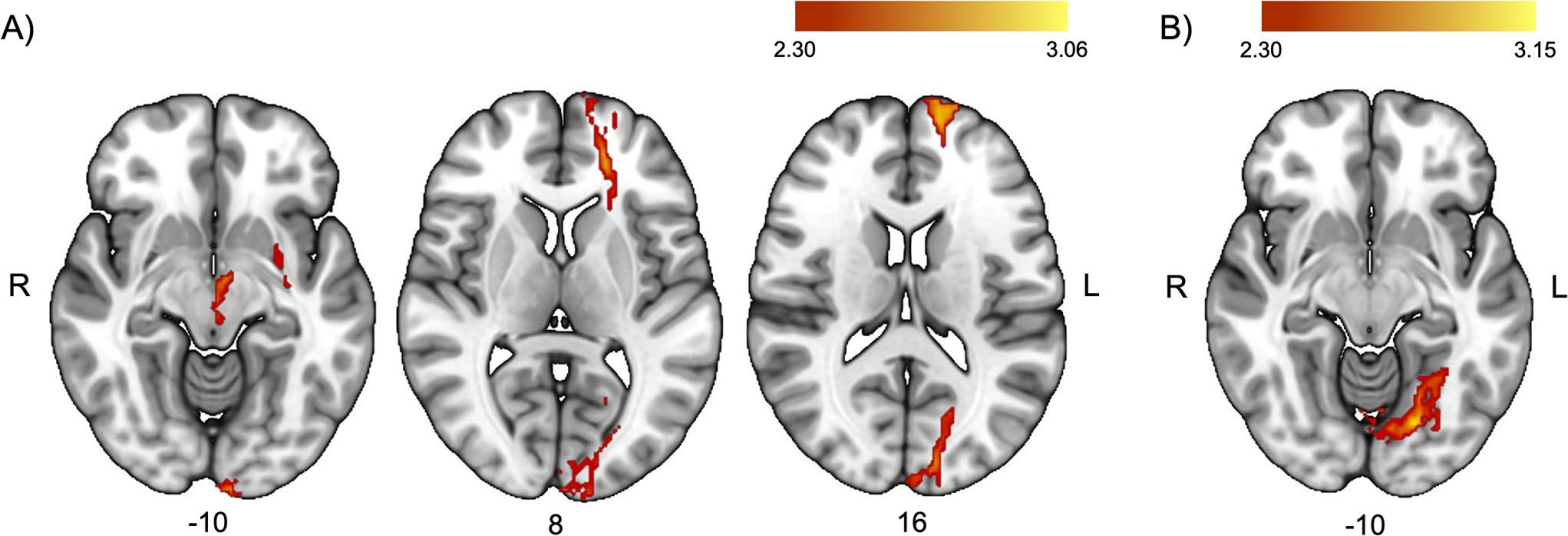
Whole-brain voxel-wise correlations between the behavioral indices of the use of attentional signals (see *methods* section for calculation method) and the contrast of interest at a cluster-defining threshold of Z > 2.3 and a corrected cluster significance threshold of *p* = 0.05 for (**A**) the alerting task and (**B**) the orienting task. The color bars denote Z-values

The whole-brain voxel-wise correlation analyses revealed significant results in an anterior cluster (*r* = 0.596, *p* = 0.009), including the white matter of the left anterior thalamic radiation (connecting midbrain areas), the left IFOF (connecting the occipital lobe), and the left uncinate (connecting the left frontal pole) and a posterior cluster (*r* = 0.502, *p* = 0.034), including the posterior part of the left IFOF. These results indicate that higher activations in these areas were associated with better use of the alerting signals (see Fig. 4A).

### Orienting task

**Fig. 5:**
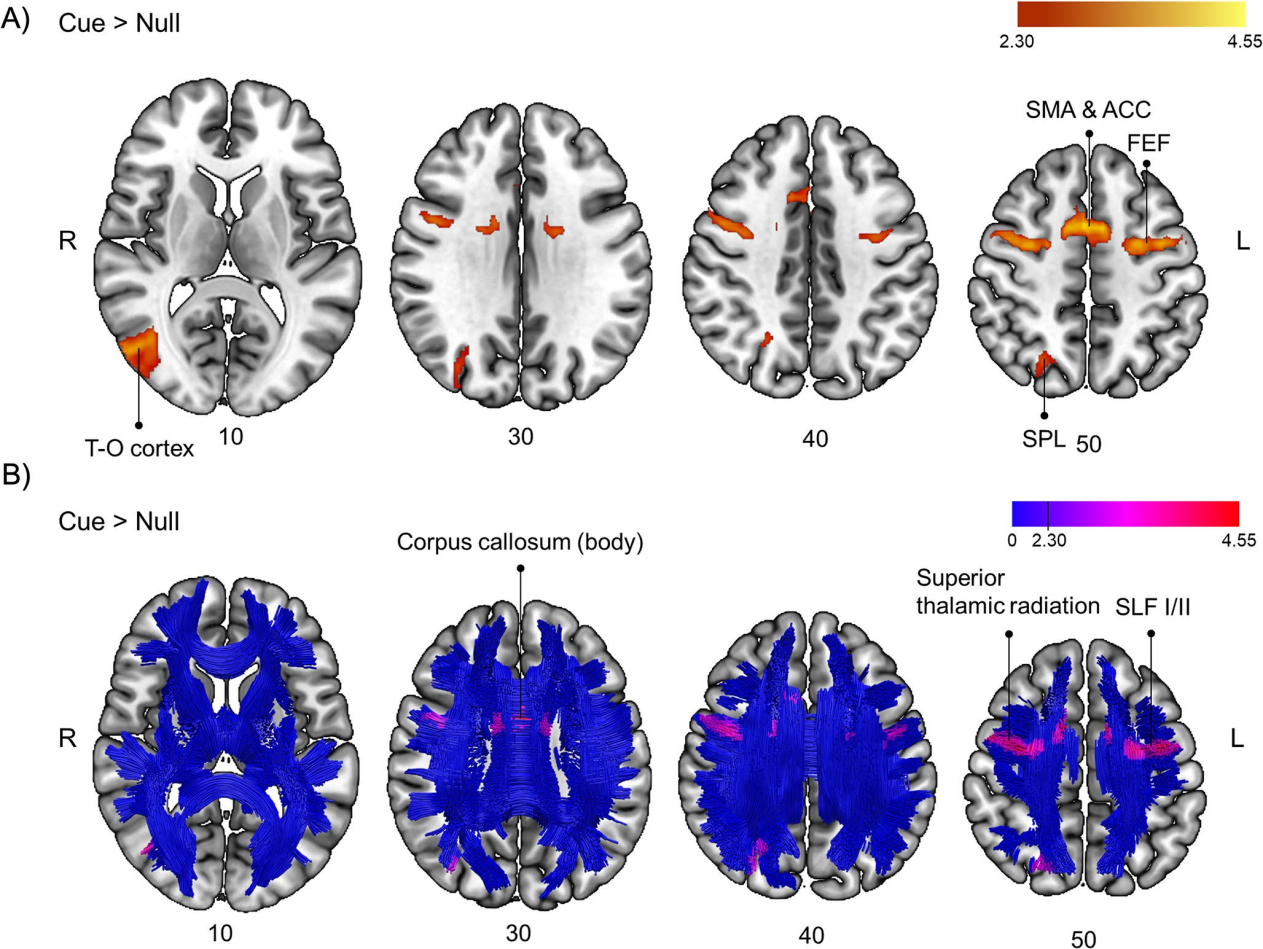
(**A**) Brain sections showing activations obtained in the contrast Cue > Null for the orienting task at a cluster-defining threshold of Z > 2.3 and a corrected cluster significance threshold of *p* = 0.05 The color bar denotes Z-values. (**B**) Representation of activations depicted in panel A over a normalized template of tractography. Tractography slices are 10 mm thick to create a 3D effect. Blue color represents the skeleton of fibers and colors from purple to red denote Z-values. ACC, anterior cingulate cortex; FEF, frontal eye field; SMA, supplementary motor area; SLF I/II, superior longitudinal fasciculus, dorsal and medial branches; SPL, superior parietal lobe; T-O cortex, temporo-occipital cortex

For the contrast Cue > Null (see Table 2; Fig. 5), we observed functional activation involving white matter association tracts running from frontal and parietal regions (the bilateral SLF I and II) and within the occipital lobe (the right vertical occipital fasciculus). Regarding projection tracts, we observed significant activations that involved the bilateral superior thalamic radiation. Finally, activations involving commissural fibers connecting frontal regions between both hemispheres (via the body of the corpus callosum) were observed. Grey matter regions involved by these connections are shown in Table 2. Additional sagittal and coronal views of the results can be found in Supplementary Fig. 2.

**Table 2:** Brain activations and connections involved in the contrast cue > null for the orienting task

Functional involvement	Side	X	Y	Z	z-value	
*White matter pathways*						*Voxels of overlap*
Corpus callosum – body	L/R	0	4	26	4.20	198 (11%)
Superior longitudinal fasciculus I	L	−24	−2	46	3.62	174 (4%)
	R	6	8	54	4.19	379 (9%)
Superior longitudinal fasciculus II	L	−26	−4	44	3.58	116 (3%)
	R	32	−4	44	3.79	139 (3%)
Superior thalamic radiation	L	−22	−4	34	3.18	225 (7%)
	R	20	−2	56	3.69	171 (5%)
Vertical occipital fasciculus	R	48	−72	0	3.65	109 (5%)
*Connections to*						*Cluster size*
Supplementary motor area (extending to anterior cingulate cortex)	L/R	2	8	54	4.55	2551 (*)
Frontal eye field	L	−42	−4	60	4.11	2551 (*)
	R	34	0	64	4.47	922
Temporooccipital cortex	R	48	−68	0	4.03	2210 (*)
Superior parietal lobe	R	20	−74	62	3.83	2210 (*)

(*) These clusters belong to a larger cluster

The whole-brain voxel-wise correlation analysis revealed a positive relation between the behavioral index and brain activation in a posterior cluster (*r* = 0.619, *p* = 0.006) including the left ILF (connecting inferior occipito-temporal areas), meaning that higher activation of these areas was associated with better use of spatial orienting cues (Fig. 4B).

### Executive attention task

**Fig. 6:**
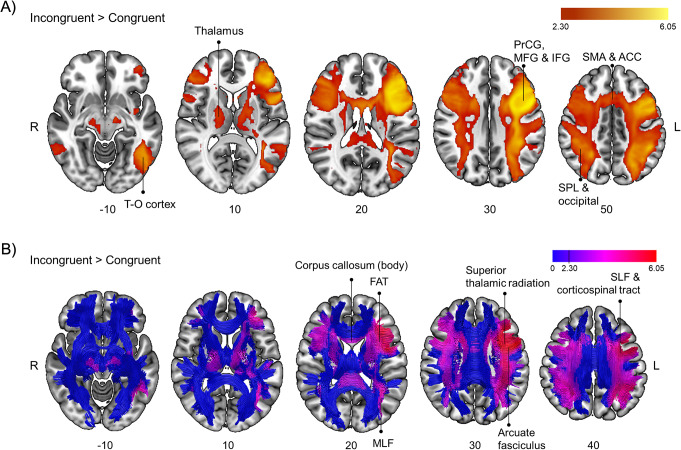
(**A**) Brain sections showing activations obtained in the contrast Incongruent > Congruent for the executive attention task at a cluster-defining threshold of Z > 2.3 and a corrected cluster significance threshold of *p* = 0.05. (**B**) Representation of activations depicted in panel A over a normalized template of tractography. Tractography slices are 10 mm thick to create a 3D effect. Blue color represents the skeleton of fibers and colors from purple to red denote Z-values. ACC, anterior cingulate cortex; FAT, frontal aslant tract; IFG, inferior frontal gyrus; MLF, middle longitudinal fasciculus; MFG, middle frontal gyrus; PrCG, precentral frontal gyrus; SLF, superior longitudinal fasciculus; SMA, supplementary motor area; SPL, superior parietal lobe; T-O cortex, temporo-occipital cortex

For the contrast Incongruent > Congruent (see Table 3; Fig. 6), we found functional activations involving 20 out of the 45 white matter tracts included in the analysis. Among them, a large amount of voxel overlap was observed involving association tracts such as the bilateral SLF (I, II, and III branches), the bilateral arcuate fasciculus, and the bilateral FAT. Considering projection tracts, the tracts showing the greatest overlap were the bilateral superior thalamic radiation and the bilateral corticospinal tract. Finally, the greatest overlaps in commissural fibers were observed in the body and the splenium of the corpus callosum. Grey matter regions involved by these connections are shown in Table 3. Additional sagittal and coronal views of the results can be found in Supplementary Fig. 3.

**Table 3:** Brain activations and connections involved in the contrast incongruent > congruent for the executive attention task

Functional involvement	Side	X	Y	Z	z-value	
*White matter pathways*						*Voxels of overlap*
Corpus callosum - body	L/R	−8	6	22	4.42	635 (37%)
Corpus callosum - splenium	L/R	−22	−48	22	3.14	458 (30%)
Acoustic radiation	L	−54	−20	6	3.71	404 (28%)
Anterior thalamic radiation	L	−28	26	18	4.96	1117 (32%)
Arcuate fasciculus	L	−36	0	26	5.85	1602 (59%)
	R	48	−36	42	4.08	1073 (34%)
Corticospinal tract	L	−34	−8	30	5.15	1507 (40%)
	R	32	−24	36	3.3	1628 (41%)
Frontal aslant tract	L	−30	16	24	5.79	2134 (84%)
	R	6	20	58	4.34	1991 (74%)
Inferior fronto-occipital fasciculus	L	−28	20	22	5.29	788 (22%)
Middle longitudinal fasciculus	L	−34	−44	24	4.68	931 (31%)
Superior longitudinal fasciculus I	L	−14	2	24	4.96	3134 (75%)
	R	26	−52	38	4.63	2645 (60%)
Superior longitudinal fasciculus II	L	−40	0	28	5.95	3630 (82%)
	R	26	−52	38	4.63	3277 (77%)
Superior longitudinal fasciculus III	L	−46	2	28	6.05	2889 (75%)
	R	38	4	26	4.23	2502 (65%)
Superior thalamic radiation	L	−30	2	28	5.76	2719 (84%)
	R	22	4	30	4.09	2434 (76%)
*Connections to*						*Cluster size*
Precentral frontal gyrus (extending to the middle and inferior frontal gyrus)	L	−46	2	28	6.05	64,472 (*)
	R	38	6	30	4.28	64,472 (*)
Superior parietal lobe	L	−48	−46	58	5.29	64,472 (*)
Superior parietal lobe (extending to the lateral occipital cortex)	R	28	−58	46	4.87	64,472 (*)
Temporo-occipital cortex	L	−52	−66	−14	4.82	64,472 (*)
	R	62	−54	−10	3.14	64,472 (*)
Supplementary motor area (extending to the anterior cingulate cortex)	L/R	0	16	54	4.63	64,472 (*)
Thalamus	L	−12	−8	8	3.34	64,472 (*)
	R	16	−16	2	3.18	64,472 (*)

(*) These clusters belong to a larger cluster

There were no significant correlations at the selected threshold between the behavioral index and brain activations.

## Discussion

In previous studies, brain function and behavior relationships have mainly been addressed by neuroimaging techniques such as fMRI, whereas brain structure and behavior associations have traditionally been assessed mainly in patients, and recently in healthy participants, by correlating grey and white matter indices with measures of healthy or altered cognitive or behavioral functioning. The present study investigated for the first time the involvement of white matter pathways in the functioning of the healthy attentional systems using a novel methodological approach. The *functionnectome* combines the signal from distant voxels of the fMRI volume using their probabilistic structural relationship given by anatomical priors derived from high-resolution tractography (Nozais et al. 2021). The aim of this study was to integrate the established knowledge regarding grey matter in attention with the relatively scarce data on white matter contributions. In addition, we aimed to link white matter and function in a more direct way, compared with traditional approaches (tract-function correlation). Our study also added new data that validate the methodology developed by Nozais and collaborators (2021) with a different cognitive process, contributing to its generalization. With these goals, we analyzed fMRI data from three different paradigms employed to manipulate alertness, spatial orienting, and executive attention, the main attentional functions that have been broadly described in the literature (Petersen and Posner 2012; Posner and Petersen 1990). By combining data that explored different attentional functions with similar experimental paradigms, and analyzing them with a new methodological approach, we aimed to get a new view of the healthy attention system that includes white matter contributions, extending previous results from the original studies.

Findings from the alerting task revealed activations in the bilateral temporal lobe, typically observed when auditory cues are employed (see e.g., Thiel and Fink 2007). Congruently, these activations involved the bilateral acoustic radiation. Tracts running from temporal regions, such as the bilateral arcuate, the left IFOF, and the bilateral MLF, were also predominantly involved. These association tracts connect the temporal lobe with other relevant areas such as occipital, parietal, and frontal regions. In addition, connections to frontal areas mainly from the left hemisphere were involved, replicating previous results (Coull et al. 2001; Fan et al. 2005). The left FAT, a short frontal connection running between the medial part of the superior frontal lobe and the IFG, was also involved. Finally, activations involving association fronto-parietal tracts such as the left SLF III were found. This set of results is not only congruent with previous findings (Clemens et al. 2011; Coull et al. 2001; Fan et al. 2005; Sturm et al. 1999; Sturm and Willmes 2001; Yanaka et al. 2010), but also offers a broad picture of the phasic alerting system. The temporal areas seem to be a crucial hub for the auditory warning system, and this area may communicate with frontal and parietal regions, mainly in the left hemisphere (as predicted), to exert its influence over the perceptual and motor systems. Indeed, connections to the left occipital and frontal areas were positively correlated with better use of the warning signals. In our main results (when we compared tone present versus tone absent trials), we did not find an involvement of midbrain or thalamic areas (Chica et al. 2016; Clemens et al. 2011; Haupt et al. 2019; Sturm et al. 1999; Sturm and Willmes 2001) or projection thalamic white matter tracts (Ge et al. 2013; Luna et al. 2021; Niogi et al. 2010), as have been found in other studies. However, when we explored the correlation between brain activation and behavior, we observed an involvement of the left anterior thalamic radiation. The absence of a group effect could be due to individual variability, that is, some of the participants did not show a greater involvement of midbrain/thalamic areas/tracts when the alerting tone was present compared with tone absent trials. Of note, those participants showing the expected midbrain/thalamic involvement also showed a larger behavioral effect (i.e., they were faster on tone present compared with tone absent trials).

Findings from the orienting task showed the expected overall right dominance for spatial orienting (Corbetta and Shulman 2002; Thiebaut de Schotten et al. 2011). They revealed an involvement of a set of fronto-parietal areas (bilateral FEFs and right SPL), showing a big overlap with the described dorsal orienting system (Corbetta and Shulman 2002), and midline regions such as the SMA and the ACC. The regions above communicate through association tracts such as the SLF I (running between the SPL and the dorsal and medial parts of the frontal lobe) and SLF II (running between the angular gyrus and the superior and middle frontal gyrus), that were involved in our study. Indeed, extensive evidence in healthy and clinical populations has related the SLF with the spatial orienting of attention (Bartolomeo et al. 2007; Carretié et al. 2012; Ciaraffa et al. 2013; Doricchi et al. 2008; Ge et al. 2013; Lunven and Bartolomeo 2017; Thiebaut de Schotten et al. 2011). In addition, we found activations in right temporo-occipital areas. Given the visual nature of the attentional cues used in this study, this is an expected result, which extends previous fMRI findings (Kincade et al. 2005). The activation of occipito-temporal regions was larger for salient non-predictive peripheral cues as compared to non-salient central predictive cues (Kincade et al. 2005). This activation may mark the cue location for the development of attentional processes in the dorsal fronto-parietal network, which includes the FEF and the parietal lobes. In our results we additionally found a positive correlation between the use of spatial orienting signals (that is, faster responses for valid compared to invalid trials) and brain activation in the left inferior occipito-temporal areas connected by the left ILF. This reinforces the idea that there is a linear relation between the activation of these regions by salient visual cues and their use in valid orienting situations. Additionally, part of the body of the corpus callosum, connecting frontal areas, was involved in our results. Involvement of the corpus callosum has been found in previous studies (Chechlacz et al. 2015; Niogi et al. 2010) and may be related with the hemispheric lateralization in spatial attention (Corbetta and Shulman 2011).

Findings from the executive attention task revealed the involvement of a broad set of brain regions. They included those traditionally implicated in executive attention (i.e., the ACC and the dlPFC, Macdonald et al. 2000; Posner and Petersen 1990) but also other frontal (e.g., IFG and SMA), parietal (e.g., SPL), temporo-occipital, and thalamic areas. Indeed, later proposals have extended the conception of “executive areas” to an executive attention network (or networks) including regions largely activated in our results (Cole and Schneider 2007; Dosenbach et al. 2008; Petersen and Posner 2012). This network conceptualization puts the focus on communication (for example, through their anatomical connections) between nodes (Zink et al. 2021). Thanks to the *functionnectome* method we were able to explore this aspect which is usually neglected in fMRI. As predicted, we found an involvement in many association tracts connecting the frontal lobe with other areas. Our results pointed to the involvement of association tracts connecting occipital and frontal areas (i.e., the left IFOF), occipital and parietal areas (i.e., left MLF), parietal and frontal areas (i.e., bilateral SLF, as expected from e.g., Crespi et al. 2018; Sasson et al. 2012, 2013; Smolker et al. 2018), temporal and frontal areas (i.e., bilateral arcuate fasciculus), and areas within the frontal lobe (i.e., FAT). In addition, our results revealed projection tracts running from the spinal cord and the thalamus to the cerebral cortex (i.e., corticospinal tract and anterior thalamic radiation) and commissural fibers (i.e., body and the splenium of the corpus callosum) connecting parietal and frontal regions in both hemispheres. This extensive set of results may reflect the complexity of executive attention processes. In addition, although we did not replicate some previous results regarding white matter tracts (i.e., anterior corona radiata, Ge et al. 2013; Niogi et al. 2010), this could be a consequence of the heterogeneity of executive attention sub-functions and the variety of paradigms and tasks employed.

These results should be interpreted in the context of some limitations of the present study. One of the limitations is the reduced sample sizes of the three analyzed studies (N between 18 and 20 participants). While the sample size was adequate to detect the expected behavioral and grey matter neural effects, additional research is warranted to validate the results concerning white matter involvement obtained through the *functionnectome* method. In addition, different experimental tasks were employed in each of the studies. Therefore, although quite similar, the paradigms had some differences beyond the attentional manipulation. In line with this, the contrasts employed for each task to isolate the attentional effects differed between studies and, by definition, in sensory conditions (e.g., the alerting task contrast compared a tone present vs. tone absent condition). This may have introduced sensory confounds in the results. Even though the aim of this study did not include comparing the results between tasks/attentional networks, this could have affected, for example, the extension of the observed brain activations or the statistical values. Additionally, we observed variations in the percentage of overlap of the results among different white matter tracts within experiments. This may be related to the functional involvement of a tract in the given attentional function. While it is premature to draw definitive conclusions from this observation, it raises an intriguing question that warrants further research. A comprehensive application of the *functionnectome* method across various paradigms and cognitive functions will enhance our conceptual understanding of the obtained results.

Overall, our results are highly consistent with the existing literature, reinforcing our knowledge of the neural bases of the healthy attentional system. The alerting network, in the context of warning signals, is sustained by temporal areas that communicate with frontal and parietal regions, mainly in the left hemisphere. The orienting network is supported by a set of bilateral fronto-parietal and midline regions communicating, within the same hemisphere, mainly via the SLF, and between hemispheres, through the corpus callosum. The complexity of executive attention processes is reflected in the implication of a broad set of brain regions and white matter tracts connecting them, supporting its conceptualization as a network or networks. Our results (i.e., involvement of the anterior thalamic radiation in the use of alerting signals, the superior thalamic radiation in orienting, and both thalamic radiations in executive attention) also converge with new evidence pointing at the importance of subcortical structures and their cortico-subcortical connections in attention (Alves et al. 2022). The equivalence of our results with previous white matter findings was somewhat less consistent. These discrepancies may be due to the relatively scarce literature on white matter, which is derived from studies with patients that link white matter lesions and cognitive deficits or from correlational studies employing DWI data and behavioral indices. These methodological differences make it difficult to directly compare previous findings with the ones obtained here. However, previous and current evidence seems to converge on the involvement of the SLF and the corpus callosum in attention processes.

In conclusion, the present study explored the functional implications of white matter in healthy attentional networks. Our results offered a more comprehensive view of the attentional system as a network of regions and their anatomical connections. They reinforce the importance of incorporating white matter within the study of human cognitive systems and of doing so in a more functional and dynamic way so as to yield a comprehensive picture of the neural bases of human cognition. Integrating the valuable information regarding white matter with well-established fMRI techniques could help to better explain and predict behavior. It would shed light on the structural pathways that connect the isolated brain regions that are usually observed in conventional fMRI studies, offering a complementary vision to the information provided by functional connectivity approaches. This network vision, supported by the structural connections, has great potential to explain how different brain regions work together to modulate behavior. It is therefore crucial to develop and extend the use of new methodologies that allow white matter functioning to be incorporated in fMRI data.

## Electronic supplementary material

Below is the link to the electronic supplementary material.

Supplementary Material 1

## Data Availability

fMRI results maps, behavioral indices, and tracts masks are publicly available via Open Science Framework (https://osf.io/xdt5j/). The conditions of the ethics approvals of the datasets do not permit public archiving or sharing of anonymized raw study data.

## Footnotes

1 In all tasks, participants underwent a titration procedure to adjust the percentage of seen targets and completed a practice block. Consequently, participants were familiar with the task, resulting in a very low percentage of both errors and missed responses across all three experiments. Specifically, the percentage of missed responses was 3.11%, 0.37%, and 2% of the trials for the alerting, orienting and executive attention tasks, respectively. The percentage of errors was 2.7% and 3.2% of the trials for the alerting and the executive attention tasks, respectively (note that the orienting task did not require participants to localize the target, thus eliminating possible response errors).

## Code availability:

Analyses were carried out using open software and toolboxes (see methods section). The functionnectome is an open-source software available at http://www.bcblab.com; all fMRI preprocesing routines and analysis were part of the FSL software tool, https://fsl.fmrib.ox.ac.uk/fsl/fslwiki/FEAT.

## References

[CR1] Alves PN, Forkel SJ, Corbetta M, De Thiebaut M (2022) The subcortical and neurochemical organization of the ventral and dorsal attention networks. Commun Biology 5(1313). 10.1038/s42003-022-04281-0 10.1038/s42003-022-04281-0 PMC9729227 36477440

[CR2] Assaf Y, Johansen-Berg H, de Thiebaut M (2019) The role of diffusion MRI in neuroscience. NMR Biomed 32(4):1–16. 10.1002/nbm.3762 10.1002/nbm.3762 28696013

[CR3] Bartolomeo P, Thiebaut de Schotten M, Doricchi F (2007) Left unilateral neglect as a disconnection syndrome. Cereb Cortex 17(11):2479–2490. 10.1093/cercor/bhl181 17272263 10.1093/cercor/bhl181

[CR4] Braver TS (2012) The variable nature of cognitive control: A dual mechanisms framework. In *Trends in Cognitive Sciences* (Vol. 16, Issue 2, pp. 106–113). Trends Cogn Sci. 10.1016/j.tics.2011.12.010 10.1016/j.tics.2011.12.010 PMC3289517 22245618

[CR5] Carretié L, Ríos M, Periáñez JA, Kessel D, Álvarez-Linera J (2012) The role of low and high spatial frequencies in exogenous attention to biologically salient stimuli. PLoS ONE 7(5):1–8. 10.1371/journal.pone.0037082 10.1371/journal.pone.0037082 PMC3349642 22590649

[CR6] Chechlacz M, Humphreys GW, Sotiropoulos SN, Kennard C, Cazzoli D (2015) Structural organization of the corpus callosum predicts attentional shifts after continuous theta burst stimulation. J Neurosci 35(46):15353–15368. 10.1523/JNEUROSCI.2610-15.2015 26586822 10.1523/JNEUROSCI.2610-15.2015 PMC4649006

[CR8] Chica AB, Paz-Alonso PM, Valero-Cabre A, Bartolomeo P (2013) Neural bases of the interactions between spatial attention and conscious perception. Cereb Cortex 23(6):1269–1279. 10.1093/cercor/bhs087 22508767 10.1093/cercor/bhs087

[CR7] Chica AB, Bayle DJ, Botta F, Bartolomeo P, Paz-Alonso PM (2016) Interactions between phasic alerting and consciousness in the fronto-striatal network. Sci Rep 6:31868. 10.1038/srep31868 27555378 10.1038/srep31868 PMC4995394

[CR9] Chica AB, Thiebaut de Schotten M, Bartolomeo P, Paz-Alonso PM (2018) White matter microstructure of attentional networks predicts attention and consciousness functional interactions. Brain Struct Function 223:653–668. 10.1007/s00429-017-1511-2 10.1007/s00429-017-1511-2 28905109

[CR10] Ciaraffa F, Castelli G, Parati EA, Bartolomeo P, Bizzi A (2013) Visual neglect as a disconnection syndrome? A confirmatory case report. Neurocase 19(4):351–359. 10.1080/13554794.2012.667130 22551209 10.1080/13554794.2012.667130

[CR11] Clemens B, Zvyagintsev M, Sack A, Heinecke A, Willmes K, Sturm W (2011) Revealing the functional neuroanatomy of intrinsic alertness using fMRI: methodological peculiarities. PLoS ONE 6(9). 10.1371/journal.pone.0025453 10.1371/journal.pone.0025453 PMC3184148 21984928

[CR12] Cole MW, Schneider W (2007) The cognitive control network: Integrated cortical regions with dissociable functions. NeuroImage 37(1):343–360. 10.1016/j.neuroimage.2007.03.071 17553704 10.1016/j.neuroimage.2007.03.071

[CR13] Corbetta M, Shulman GL (2002) Control of goal-Directed and stimulus-driven attention in the brain. Nat Rev Neurosci 3(3):215–229. 10.1038/nrn755 10.1038/nrn755 11994752

[CR61] Corbetta M, Patel G, Shulman GL. (2008). The reorienting system of the human brain: from environment to theory of mind. Neuron 58(3):306–324. 10.1016/j.neuron.2008.04.017 10.1016/j.neuron.2008.04.017 PMC2441869 18466742

[CR14] Corbetta M, Shulman GL (2011) Spatial neglect and attention networks. Annu Rev Neurosci 34:569–599. 10.1146/annurev-neuro-061010-113731 21692662 10.1146/annurev-neuro-061010-113731 PMC3790661

[CR15] Coull JT, Nobre AC, Frith CD (2001) The noradrenergic α2 agonist clonidine modulates behavioural and neuroanatomical correlates of human attentional orienting and alerting. Cereb Cortex 11(1):73–84. 10.1093/cercor/11.1.73 11113036 10.1093/cercor/11.1.73

[CR16] Crespi C, Laureiro-Martínez D, Dodich A, Cappa SF, Brusoni S, Zollo M, Falini A, Canessa N (2018) Improving innovative decision-making: training-induced changes in fronto-parietal networks. Brain Cogn 128:46–55. 10.1016/J.BANDC.2018.11.004 30468942 10.1016/J.BANDC.2018.11.004

[CR17] Dale AM (1999) Optimal experimental design for event-related fMRI. Hum Brain Mapp 8(2–3):109–114. 10.1002/(SICI)1097-0193(1999)8:2/3<109::AID-HBM7%3.0.CO;2-W 10524601 10.1002/(SICI)1097-0193(1999)8:2/3<109::AID-HBM7>3.0.CO;2-W PMC6873302

[CR18] Dell’Acqua, Simmons A, Williams SCR, Catani M (2013) Can spherical deconvolution provide more information than fiber orientations? Hindrance modulated orientational anisotropy, a true-tract specific index to characterize white matter diffusion. Hum Brain Mapp 34(10):2464–2483. 10.1002/hbm.22080 22488973 10.1002/hbm.22080 PMC6870506

[CR19] Doricchi F, Thiebaut de Schotten M, Tomaiuolo F, Bartolomeo P (2008) White matter (dis)connections and gray matter (dys)functions in visual neglect: gaining insights into the brain networks of spatial awareness. Cortex 44(8):983–995. 10.1016/j.cortex.2008.03.006 18603235 10.1016/j.cortex.2008.03.006

[CR20] Dosenbach NUF, Fair DA, Cohen AL, Schlaggar BL, Petersen SE (2008) A dual-networks architecture of top-down control. Trends Cogn Sci 12(3):99–105. 10.1016/j.tics.2008.01.001 18262825 10.1016/j.tics.2008.01.001 PMC3632449

[CR21] Fan J, McCandliss BD, Fossella J, Flombaum JI, Posner MI (2005) The activation of attentional networks. NeuroImage 26(2):471–479. 10.1016/j.neuroimage.2005.02.004 15907304 10.1016/j.neuroimage.2005.02.004

[CR22] Forkel SJ, Friedrich P, Thiebaut de Schotten M, Howells H (2022) White matter variability, cognition, and disorders: a systematic review. Brain Struct Function 227(2):529–544. 10.1007/s00429-021-02382-w 10.1007/s00429-021-02382-w PMC8844174 34731328

[CR23] Gawryluk JR, Mazerolle EL, D’Arcy RCN (2014) Does functional MRI detect activation in white matter? A review of emerging evidence, issues, and future directions. Front NeuroSci 8(239):1–12. 10.3389/fnins.2014.00239 25152709 10.3389/fnins.2014.00239 PMC4125856

[CR24] Ge H, Yin X, Xu J, Tang Y, Han Y, Xu W, Pang Z, Meng H, Liu S (2013) Fiber pathways of attention subnetworks revealed with tract-based spatial statistics (TBSS) and probabilistic tractography. PLoS ONE 8(11):1–7. 10.1371/journal.pone.0078831 10.1371/journal.pone.0078831 PMC3817088 24223852

[CR25] Gore JC, Li M, Gao Y, Wu TL, Schilling KG, Huang Y, Mishra A, Newton AT, Rogers BP, Chen LM, Anderson AW, Ding Z (2019) Functional MRI and resting state connectivity in white matter - a mini-review. Magn Reson Imaging 63:1–11. 10.1016/j.mri.2019.07.017 31376477 10.1016/j.mri.2019.07.017 PMC6861686

[CR26] Haupt M, Ruiz-Rizzo AL, Sorg C, Finke K (2019) Phasic alerting effects on visual processing speed are associated with intrinsic functional connectivity in the cingulo-opercular network. NeuroImage 196:216–226. 10.1016/j.neuroimage.2019.04.019 30978493 10.1016/j.neuroimage.2019.04.019

[CR27] Jenkinson M, Bannister P, Brady M, Smith S (2002) Improved optimization for the robust and accurate linear registration and motion correction of brain images. NeuroImage 17(2):825–841. 10.1016/S1053-8119(02)91132-8 12377157 10.1016/S1053-8119(02)91132-8

[CR28] Kardan O, Stier AJ, Cardenas-Iniguez C, Schertz KE, Pruin JC, Deng Y, Chamberlain T, Meredith WJ, Zhang X, Bowman JE, Lakhtakia T, Tindel L, Avery EW, Lin Q, Yoo K, Chun MM, Berman MG, Rosenberg MD (2022) Differences in the functional brain architecture of sustained attention and working memory in youth and adults. PLoS Biol 20(12):e3001938. 10.1371/JOURNAL.PBIO.3001938 36542658 10.1371/JOURNAL.PBIO.3001938 PMC9815648

[CR29] Kincade JM, Abrams RA, Astafiev SV, Shulman GL, Corbetta M (2005) An event-related functional magnetic resonance imaging study of voluntary and stimulus-driven orienting of attention. J Neurosci 25(18):4593–4604. 10.1523/JNEUROSCI.0236-05.2005 15872107 10.1523/JNEUROSCI.0236-05.2005 PMC6725019

[CR30] Le Bihan D, Mangin J-F, Poupon C, Clark CA, Pappata S, Molko N, Chabriat H (2001) Diffusion tensor imaging: concepts and applications. J Magn Reson Imaging 13(4):534–546. 10.1002/jmri.1076 11276097 10.1002/jmri.1076

[CR31] Luna FG, Lupiáñez J, Martín-Arévalo E (2021) Microstructural white matter connectivity underlying the attentional networks system. Behav Brain Res 401(113079):1–12. 10.1016/j.bbr.2020.113079 10.1016/j.bbr.2020.113079 33358923

[CR32] Lunven M, Bartolomeo P (2017) Attention and spatial cognition: neural and anatomical substrates of visual neglect. Annals Phys Rehabilitation Med 60:124–129. 10.1016/j.rehab.2016.01.004 10.1016/j.rehab.2016.01.004 26874577

[CR33] Macdonald AW, Cohen JD, Stenger VA, Carter CS (2000) Dissociating the role of the Dorsolateral Prefrontal and Anterior Cingulate Cortex in cognitive control. Science 288:1835–1838. 10.1126/science.288.5472.1835 10846167 10.1126/science.288.5472.1835

[CR34] Martín-Signes M, Paz-Alonso PM, Chica AB (2019) Connectivity of Frontoparietal regions reveals executive attention and consciousness interactions. Cereb Cortex 29(11):4539–4550. 10.1093/cercor/bhy332 30590403 10.1093/cercor/bhy332

[CR35] Miller EK, Cohen JD (2001) An integrative theory of Prefrontal cortex function. Annu Rev Neurosci 24(1):167–202. 10.1146/annurev.neuro.24.1.167 11283309 10.1146/annurev.neuro.24.1.167

[CR36] Mori S, Wakana S, Van Zijl PCM, Nagae-Poetscher LM (2005) MRI atlas of human white matter. Elsevier. 10.1002/cmr.a.20051

[CR37] Niogi S, Mukherjee P, Ghajar J, McCandliss BD (2010) Individual differences in distinct components of attention are linked to anatomical variations in distinct white matter tracts. Front Neuroanat 4:1–12. 10.3389/neuro.05.002.2010 20204143 10.3389/neuro.05.002.2010 PMC2831631

[CR38] Norman DA, Shallice T (1986) Attention to action: willed and automatic control of behaviour. In: Davidson RJ, Schwartz GE, Shapiro D (eds) Consciousness and self-regulation. Springer US, pp 1–18. 10.1007/978-1-4757-0629-1_1

[CR39] Nozais V, Forkel SJ, Foulon C, Petit L, de Thiebaut M (2021) Functionnectome: a framework to analyse the contribution of brain circuits to fMRI. Commun Biology 1035. 10.1038/s42003-021-02530-2 10.1038/s42003-021-02530-2 PMC8413369 34475518

[CR40] Petersen SE, Posner MI (2012) The attention system of the human brain: 20 years after. Annu Rev Neurosci 21(35):73–89. 10.1146/annurev-neuro-062111-150525 10.1146/annurev-neuro-062111-150525 PMC3413263 22524787

[CR41] Posner MI (1980) Orienting of attention. Q J Experimental Psychol 32(1):3–25. 10.1080/00335558008248231 10.1080/00335558008248231 7367577

[CR42] Posner MI (1994) Attention: the mechanisms of consciousness. Proc Natl Acad Sci USA 91:7398–7403. 10.1073/pnas.91.16.7398 8052596 10.1073/pnas.91.16.7398 PMC44408

[CR43] Posner MI, Petersen SE (1990) The attention system of the human brain. Annu Rev Neurosci 13:25–42. 10.1146/annurev-neuro-062111-150525 2183676 10.1146/annurev-neuro-062111-150525

[CR44] Rosenberg MD, Finn ES, Scheinost D, Constable RT, Chun MM (2017) Characterizing attention with Predictive Network models. Trends Cogn Sci 21(4):290–302. 10.1016/J.TICS.2017.01.011 28238605 10.1016/J.TICS.2017.01.011 PMC5366090

[CR45] Sasson E, Doniger GM, Pasternak O, Tarrasch R, Assaf Y (2012) Structural correlates of cognitive domains in normal aging with diffusion tensor imaging. Brain Struct Function 217(2):503–515. 10.1007/s00429-011-0344-7 10.1007/s00429-011-0344-7 21909706

[CR46] Sasson E, Doniger GM, Pasternak O, Tarrasch R, Assaf Y (2013) White matter correlates of cognitive domains in normal aging with diffusion tensor imaging. Front NeuroSci 7(32):1–13. 10.3389/fnins.2013.00032 23493587 10.3389/fnins.2013.00032 PMC3595518

[CR47] Smith SM (2002) Fast robust automated brain extraction. Hum Brain Mapp 17(3):143–155. 10.1002/hbm.10062 12391568 10.1002/hbm.10062 PMC6871816

[CR48] Smolker HR, Friedman NP, Hewitt JK, Banich MT (2018) Neuroanatomical correlates of the Unity and Diversity Model of Executive Function in young adults. Front Hum Neurosci 12:283. 10.3389/fnhum.2018.00283 30083098 10.3389/fnhum.2018.00283 PMC6064948

[CR59] Statements & Declarations

[CR50] Sturm W, Willmes K (2001) On the functional neuroanatomy of intrinsic and phasic alertness. NeuroImage 14:76–84. 10.1006/nimg.2001.0839 10.1006/nimg.2001.0839 11373136

[CR49] Sturm W, De Simone A, Krause BJ, Specht K, Hesselmann V, Radermacher I, Herzog H, Tellmann L, Müller-Gärtner HW, Willmes K (1999) Functional anatomy of intrinsic alertness: evidence for a fronto-parietal-thalamic-brainstem network in the right hemisphere. Neuropsychologia 37(7):797–805. 10.1016/S0028-3932(98)00141-9 10408647 10.1016/S0028-3932(98)00141-9

[CR51] Thiebaut de Schotten M, Dell’Acqua F, Forkel SJ, Simmons A, Vergani F, Murphy DGM, Catani M (2011) A lateralized brain network for visuospatial attention. Nat Neurosci 14(10):1245–1247. 10.1038/nn.2905 21926985 10.1038/nn.2905

[CR60] Thiel CM, Zilles K, Fink GR (2004) Cerebral correlates of alerting, orienting and reorienting of visuospatial attention: an event-related fMRI study. Neuroimage 21(1):318–328. 10.1016/j.neuroimage.2003.08.044 10.1016/j.neuroimage.2003.08.044 14741670

[CR52] Thiel CM, Fink GR (2007) Visual and auditory alertness: modality-specific and supramodal neural mechanisms and their modulation by nicotine. J Neurophysiol 97(4):2758–2768. 10.1152/JN.00017.2007 17287445 10.1152/JN.00017.2007

[CR53] Warrington S, Bryant KL, Khrapitchev AA, Sallet J, Charquero-Ballester M, Douaud G, Jbabdi S, Mars RB, Sotiropoulos SN (2020) XTRACT - standardised protocols for automated tractography in the human and macaque brain. NeuroImage 217:116923. 10.1016/j.neuroimage.2020.116923 32407993 10.1016/j.neuroimage.2020.116923 PMC7260058

[CR55] Woolrich MW, Ripley BD, Brady M, Smith SM (2001) Temporal autocorrelation in univariate linear modeling of FMRI data. NeuroImage 14(6):1370–1386. 10.1006/nimg.2001.0931 11707093 10.1006/nimg.2001.0931

[CR54] Woolrich MW, Behrens TEJ, Beckmann CF, Jenkinson M, Smith SM (2004) Multilevel linear modelling for FMRI group analysis using bayesian inference. NeuroImage 21(4):1732–1747. 10.1016/j.neuroimage.2003.12.023 15050594 10.1016/j.neuroimage.2003.12.023

[CR56] Yanaka HT, Saito DN, Uchiyama Y, Sadato N (2010) Neural substrates of phasic alertness: a functional magnetic resonance imaging study. Neurosci Res 68(1):51–58. 10.1016/j.neures.2010.05.005 20561955 10.1016/j.neures.2010.05.005

[CR57] Yoo K, Rosenberg MD, Kwon YH, Lin Q, Avery EW, Sheinost D, Constable RT, Chun MM (2022) A brain-based general measure of attention. Nat Hum Behav 6(6):782. 10.1038/S41562-022-01301-1 35241793 10.1038/S41562-022-01301-1 PMC9232838

[CR58] Zink N, Lenartowicz A, Markett S (2021) A new era for executive function research: on the transition from centralized to distributed executive functioning. Neurosci Biobehavioral Reviews 124:235–244. 10.1016/j.neubiorev.2021.02.011 10.1016/j.neubiorev.2021.02.011 PMC8420078 33582233

