# Supplementary material for: Integrating brain function and structure in the study of the human attentional networks: a functionnectome study
Source: Brain Struct Funct. 2024 Jul 6;229(7):1665–79. doi: 10.1007/s00429-024-02824-1 (PMC11374869; doi:10.1007/s00429-024-02824-1)
Supplement: Supplementary file 1 — Supplementary Material 1 [file 429_2024_2824_MOESM1_ESM.docx]

**Supplementary Figures**
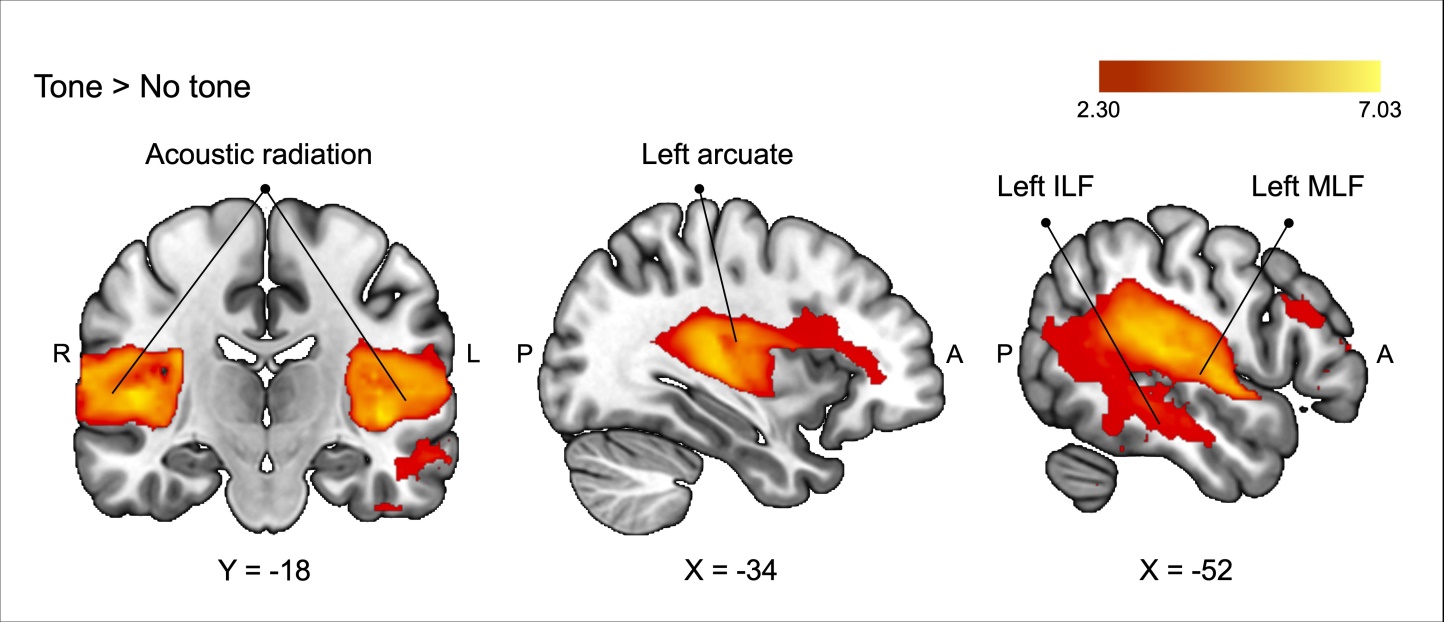


**Supplementary Figure 1.** Additional brain sections showing activations and white matter involvement obtained in the contrast Tone > No tone for the alerting task at a statistical Z-threshold of >2.3 and a corrected cluster significance threshold of p = 0.05. The color bar denotes Z-values. ILF, inferior longitudinal fasciculus; MLF, middle longitudinal fasciculus.


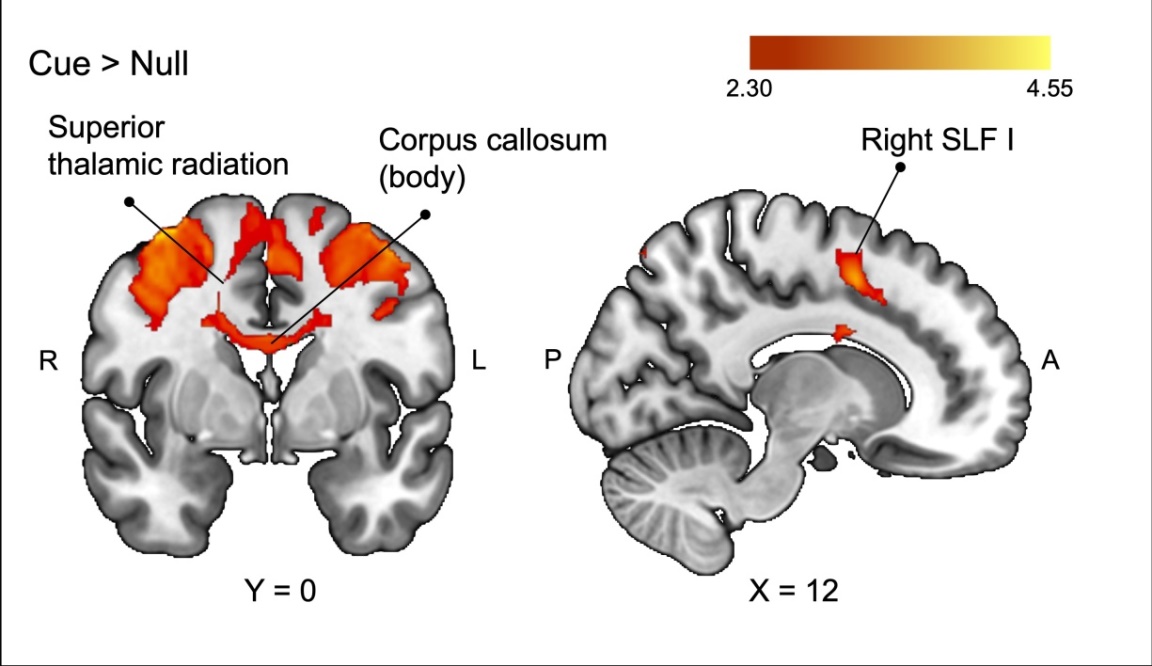


**Supplementary Figure 2.** Additional brain sections showing activations and white matter involvement obtained in the contrast Cue > Null for the orienting task at a statistical Z-threshold of >2.3 and a corrected cluster significance threshold of p = 0.05. The color bar denotes Z-values. SLF, superior longitudinal fasciculus.


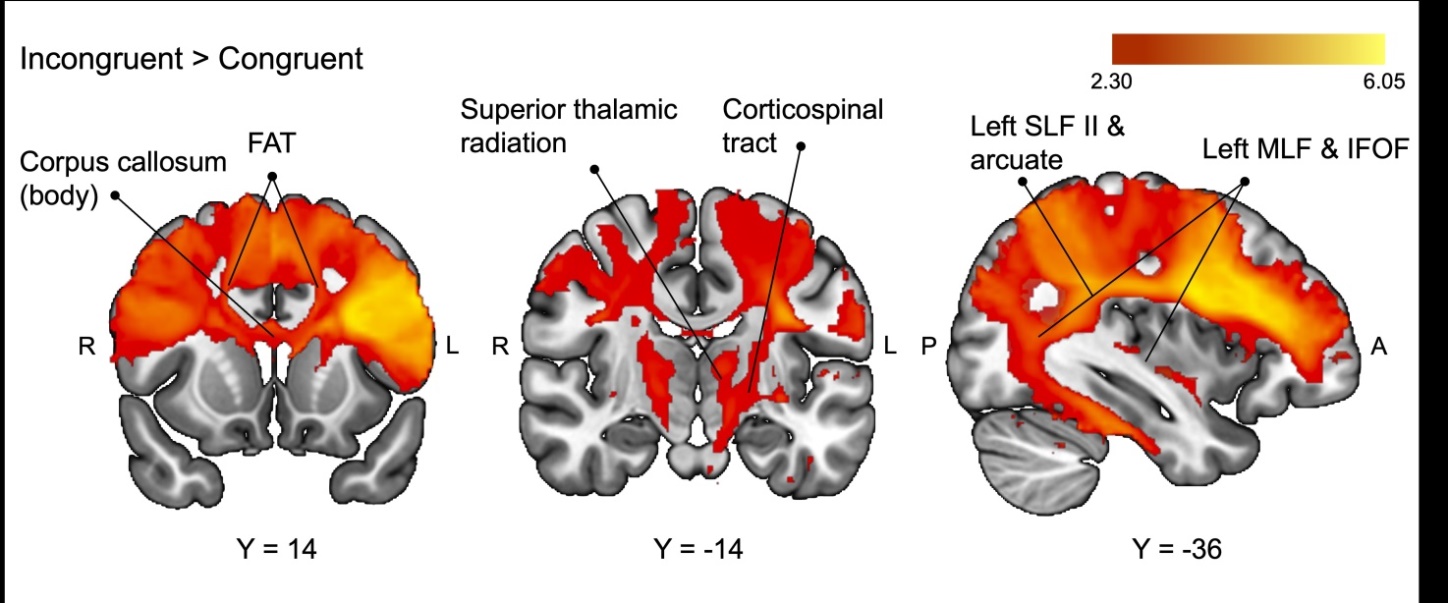


**Supplementary Figure 3.** Additional brain sections showing activations and white matter involvement obtained in the contrast Incongruent > Congruent for the executive attention task at a statistical Z-threshold of >2.3 and a corrected cluster significance threshold of p = 0.05. The color bar denotes Z-values. FAT, frontal aslant tract; IFOF, inferior frontal-occipital fasciculus; MLF, middle longitudinal fasciculus; SLF, superior longitudinal fasciculus.
